# Physicochemical Characteristics and Occupational Exposure of Silica Particles as Byproducts in a Semiconductor Sub Fab

**DOI:** 10.3390/ijerph19031791

**Published:** 2022-02-04

**Authors:** Kwang-Min Choi, Soo-Jin Lee

**Affiliations:** 1 Samsung Health Research Institute, Environment & Safety Center, Samsung Electronics Co., Ltd., Hwaseong-si 18448, Korea; 2 Department of Occupational and Environmental Medicine, College of Medicine, Hanyang University, Seoul 04763, Korea

**Keywords:** amorphous silica, nanoparticles, particulate matter, byproduct, chemical vapor deposition, diffusion, semiconductor fabrication facility

## Abstract

This study aimed to elucidate the physicochemical characteristics and occupational exposure of silica powder and airborne particles as byproducts generated from the first scrubbers of chemical vapor deposition and diffusion processes during maintenance in a semiconductor facility sub fab to reduce unknown risk factors. The chemical composition, size, morphology, and crystal structure of powder and airborne particles as byproducts were investigated using a scanning electron microscopy and transmission electron microscopy equipped with an energy dispersive X-ray spectroscopy, and an X-ray diffraction. The number and mass concentration measurements of airborne particles were performed by using an optical particle sizer of a direct-reading aerosol monitor. All powder and airborne particle samples were mainly composed of oxygen (O) and silicon (Si), which means silica. The byproduct particles were spherical and/or nearly spherical and the particle size ranged from 10 to 90 nm, based on primary particles. Most of the particles were usually agglomerated within a particle size range from approximately 100 nm to 35 µm. In addition, most of the powder samples exhibited diffraction patterns with a broad and relatively low intensity at 2θ degrees 21.6–26.7°, which is similar to that of pure amorphous silica. The above results show the byproduct particles are amorphous silica, which are considered a less toxic foam compared to crystalline silica. The number and mass concentrations of PM10 (particles less than 10 µm in diameter) ranged from 4.250–78.466 particles/cm^3^ and 0.939–735.531 µg/m^3^, respectively. In addition, 0.3–1.0 and 2.5–10 µm particles occupied the highest portion of the number and mass concentrations, respectively. Meanwhile, several peak exposure patterns were observed at a specific step, which is the process of removing powder particles on the inner chamber and cleaning the chamber by using a vacuum cleaner and a clean wiper, during the maintenance task.

## 1. Introduction

The semiconductor industry is one of the fastest growing and most rapidly changing manufacturing sectors in the world. The demand for novel, diverse and complicated chemical substances is increasing, according to the shrinkage of the design rules [1,2,3]. To produce semiconductors, an extremely well-controlled and clean environment is needed, and must be maintained at the correct temperature, humidity and pressurization of the clean room, and provide forced air circulation. Workers, including the operator, and the process and facilities engineers, in the clean room must be covered from head to toe in dust-free garments, to protect against the spread of contaminant particles [4]. Meanwhile, almost all items of production equipment in the semiconductor industry are closed in operation, and the excess chemicals used in the manufacturing process are removed by exhaust ventilation systems. In addition, non-reactive residual materials (or waste gases) and process byproducts, which exist inside the reaction chambers, are removed through an in situ cleaning process using nitrogen trifluoride (NF_3_) and chlorine trifluoride (ClF_3_) with plasma and/or heat, to prevent process and product defects in advance [5,6]. The waste gasses remaining to be used in the semiconductor manufacturing processes are usually discharged through the first and second scrubber treatments to meet air pollution emission limits. However, despite the use of exhaust ventilation and cleaning systems, it is impossible to completely remove the waste gases and byproducts from the equipment inside. These non-reactive residual chemicals and/or byproducts can result in worker exposure and inhalation during the maintenance of production equipment and the first scrubber, and the health impact of the workers in the workplace should be considered.

Generally, the semiconductor manufacturing process can be divided into wafer fabrication and assembly. The wafer fabrication is to produce the semiconductor integrated circuits, which includes various unit operations, such as photolithography, dry etching, wet cleaning, metallization, chemical vapor deposition (CVD), diffusion, ion implantation and chemical mechanical polishing [1,3]. The entire fabrication process consists of 500 to 700 steps, according to the specific semiconductor device; most devices require multiple steps through the same processes, at different stages. Even after the fabrication process is completed, it is made into a complete product through the assembly process, including sawing, die attachment, wire bonding, molding, and testing [7,8,9]. The assembly process is key to assuring the reliability and quality of the semiconductor products, that is, it is to protect the electronic components from an adverse environment and mechanical shock, and to provide structural support and electrical insulation.

Since the wafer surface is composed of Si, it easily reacts with oxygen in the air to produce silica (SiO_2_) of a native oxide film. In addition, SiO_2_ film having excellent insulating properties can be made by reacting various Si-contained precursors with oxygen at a high temperature. Therefore, SiO_2_ film is one of the most important films in the semiconductor fabrication process [10]. For these reasons, in the semiconductor fabrication process, silicon dioxide (silica, SiO_2_) can be produced as a process byproduct, and can be expected to be exposed to the air in the workplace during the maintenance of production equipment and the first scrubber. Generally, silica is classified as crystalline and amorphous silica, according to the crystal structure. It is well known that health effects of silica are dependent on the crystal structure [11,12,13]. Crystalline silica may cause both acute and chronic pulmonary inflammatory responses and are a known carcinogen, while less is known about the toxicity of amorphous silica, with no studies having classified amorphous silica with regard to its carcinogenicity to humans [13,14,15]. Recently, the toxicity of some silica nanoparticles has been reported, but the toxicity effect is still unclear [16,17,18]. In addition, it is known that the inhalation toxicity varies depending on the particle size and shape [19,20]. For these reasons, identification research on the size, shape, and crystal structure of silica particles that can be exposed to workers must be carried out from the point of view of worker health in the working environment. However, a detailed analysis of the physicochemical properties of Si-contained powder and airborne byproducts generated from the first scrubbers in a semiconductor facility sub fab, abbreviated as FS-FAB, has not been carried out; thus, there is a need for the study of these powders to reduce uncertainty about the potential harmful factors in the working environment. Therefore, the aim of this study is to identify the physicochemical properties that include the elemental component, size, morphology, and crystal structure of the powder and airborne byproducts that would be generated and/or released from the first scrubber of the chemical vapor deposition and diffusion processes, which is predicted to be produced silica as byproducts in the semiconductor FS-FAB.

## 2. Materials and Methods

### 2.1. Sampling Site and Target Process

This study was conducted in a semiconductor fabrication facility, that produce 300 mm wafers. Herein, a 300 mm wafer fabrication facility is generally divided into a clean room (or FAB), clean sub fab (CS-FAB) and facility sub fab (FS-FAB) [21]. The sampling sites were a FS-FAB, which provides equipment to process the chemicals needed for wafer fabrication and houses accessory equipment, such as pump and first scrubber for the treatment and exhaustion of waste gases. Chemical vapor deposition (CVD); detail step—silicon nitride (Si_3_N_4_), and diffusion; detail step—poly Si and SiO_2_ film deposition were selected as target processes, because silica powder byproducts are capable of being generated from the processes.

### 2.2. Powder and Airborne Particle Sample Collection

All eight powder byproduct samples [CVD (3) and diffusion (5)] were collected from the inner chamber parts during first scrubber maintenance, including cleaning (removal of powder particles and reactant residues) and replacement of chamber and other parts. The procedure of powder sample collection is as follows: (a) stop the operation of the scrubber > (b) take the chamber from the scrubber > (c) collect the powder inside the chamber. The powder sample was collected directly using a clean wiper used for semiconductor process and a polypropylene conical centrifuge tube (352098, Falcon, Becton-Dickinson, Franklin Lakes, NJ, USA). In addition, airborne particle samples were collected by airborne area sampling, which was performed for 30 min at a flow rate of 2.0 L/min, using pre- and post- calibrated air sampling pumps (GirAir3, Gilian, Sensidyne Inc., Clearwater, FL, USA) connected with a preloaded polyvinylchloride (PVC) membrane filter (pore size 5.0 µm, diameter 37 mm) in a 3-piece 37 mm cassette (255-803, SKC Inc. Eighty Four, PA, USA).

### 2.3. Maintenance Task and Personal Protective Equipment

The maintenance task of first scrubber is carried out in the following sequence: (step 1) set a dust-free pad at the bottom of front door of scrubber (elapse time: 1–2 min), (step 2) after opening the front door and removing the scrubber chamber, put the chamber in a dust-free plastic bag (elapse time: 5–8 min), (step 3) remove the powder particles in the scrubber and duct by using a vacuum cleaner (elapse time: 15–20 min), (step 4) remove the powder particles on the inside and outside surface of the scrubber chamber by using the vacuum cleaner and a clean wiper (elapse time: 25–30 min), (step 5) assemble the cleaned chamber and close the front door to finish (elapse time: 30–35 min). The number of workers per one first scrubber is two, and the total maintenance time is approximately 30 min. During maintenance, workers wear personal protection equipment, such as a safety helmet, safety shoes, dust-free polyvinyl chloride or nitrile gloves, acid resistance gloves, apron, wristlets, safety goggles, and 3M 7502 half-facepiece respirators with 3M 6006K (multi gases-vapor cartridge) and 3M particulate filter 5N11, N95.

### 2.4. Airborne PM Concentration and Size Distribution

The number and mass concentration and size distribution of airborne particulate matter (PM) were determined by optical particle sizer (OPS, TSI 3330, TSI Inc., Shoreview, MN, USA), which is capable of counting particle sizes in three size ranges from 0.3 to 10 µm, i.e., 0.3–1.0, 1.0–2.5, and 2.5–10 µm, for 20–30 min at a flow rate of 1.0 L/min, during maintenance of first scrubber. The data of number and mass concentrations of airborne particles were taken once per five seconds. The detection limits of the number and mass concentration of the OPS are 0.001 particles/cm^3^ and 0.001 µg/m^3^, respectively. To approximate the conditions of exposure, all airborne PM measurements and samplings were conducted within 0.2–0.5 m from each item of first scrubber at about 0.8–1.0 m above floor level during maintenance (Supplementary Material Figure S1).

### 2.5. Physicochemical Characterization

The chemical composition, size, and morphology of the powder and airborne samples were determined by scanning electron microscopy (SEM, JSM 7001F, JEOL, Tokyo, Japan) and transmission electron microscopy (TEM, TITAN 30-800, FEI Inc., Hillsboro, OR, USA) equipped with energy dispersive X-ray spectroscopy (EDS, INCA 200, Oxford Instruments, Abingdon, Oxfordshire, UK). SEM analysis conditions are as follows: accelerating voltage: 20 kV; magnification: 1000–10,000 × magnification; observation field number: 5. The powder samples were placed on conductive adhesive tape composed of carbon and observed, and the filters (which collected airborne particles) were coated with gold (Au) before analysis. In addition, TEM analysis conditions are as follows: accelerating voltage: 300 kV; magnification: 10,000–350,000 × magnification; observation field number: 5. Crystal structure of the powder sample was analyzed by X-ray diffraction (XRD, D8 Advance, Bruker AXS GmbH, Karlsruhe, Germany). For the XRD analysis, the powder sample was deposited in the powder holder, and the analysis was carried out over the range of diffraction angles of 10 to 60°, in steps of 0.02°. The database used for indexing the diffraction pattern was Powder Diffraction File 2 (Version 4.1) of the International Center for Diffraction Data (Swarthmore, PA, USA). To perform comparative analysis of the crystal structure of the powder byproduct, the amorphous and crystalline silicon dioxide (SiO_2_) powders (99.9% up purity, Kojundo Chemical Laboratory Co. Ltd., Saitama, Japan) were also analyzed by XRD.

## 3. Results

### 3.1. Chemical Composition

Table 1 shows the elemental components of the powder and airborne particles as byproducts collected from the first scrubbers of the CVD and diffusion processes during maintenance, and the chemicals used in the processes (Supplementary Material Figure S2). Meanwhile, nitrogen trifluoride (NF_3_) was used for the cleaning production equipment chamber, and no gas or chemical was used in the first scrubber, but only liquefied natural gas and water. For the scrubber of CVD silicon nitride (Si_3_N_4_), all powder and airborne particle samples were mainly composed of oxygen (O) and silicon (Si), which means silica particles, and also fluorine (F) elements were intermittently detected according to the sample. On the other hand, for the diffusion, O and Si were detected in common, and F, Ni, Na, Mg, Al, K, and Ca elements were also intermittently detected.

### 3.2. Size and Surface Morphology

**Figure 1:**
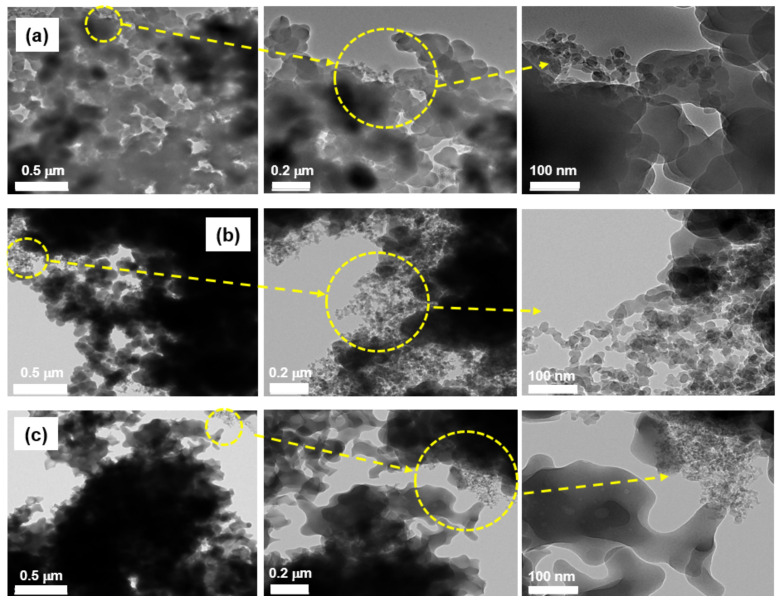
Transmission electron microscopy images of the powder byproducts collected inside the reaction chambers during the first scrubber maintenance of chemical vapor deposition for Si_3_N_4_ film deposition; (**a**) sample 1, (**b**) sample 2, (**c**) sample 3.

**Figure 2:**
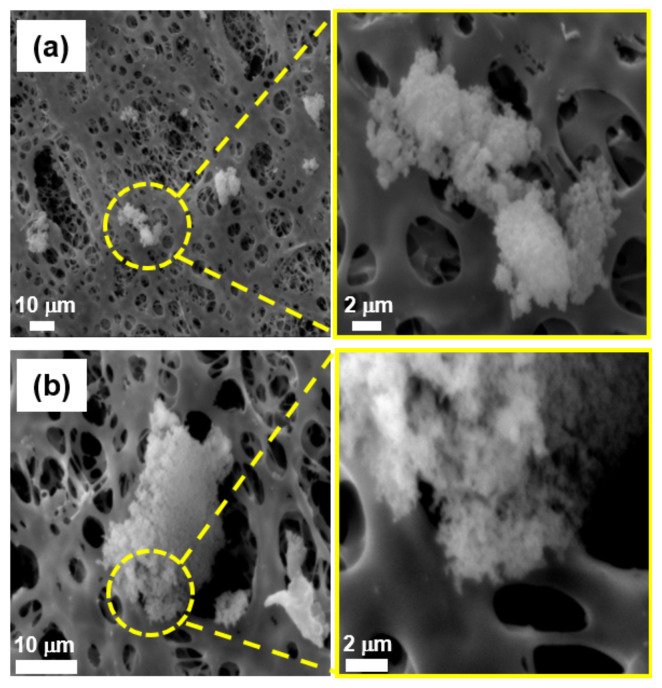
Scanning electron microscopy images of the airborne particles as byproducts in the first scrubber maintenance of chemical vapor deposition process for Si_3_N_4_ film deposition; (**a**) sample 2, (**b**) sample 3.

**Figure 3:**
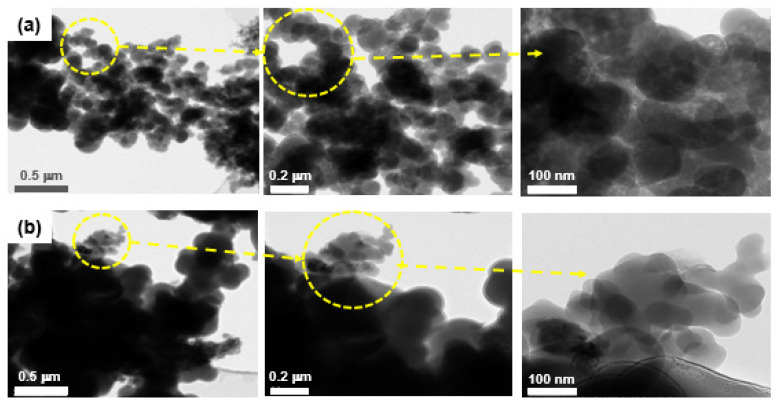

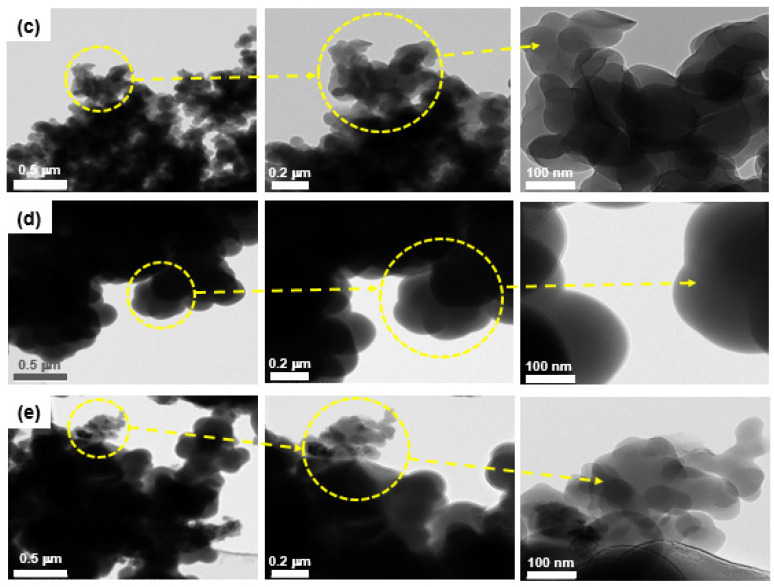
Transmission electron microscopy images of the powder byproducts collected inside the reaction chambers during the first scrubber maintenance of diffusion process for poly Si and SiO_2_ deposition; (**a**) sample 4, (**b**) sample 5, (**c**) sample 6, (**d**) sample 7, (**e**) sample 8.

**Figure 4:**
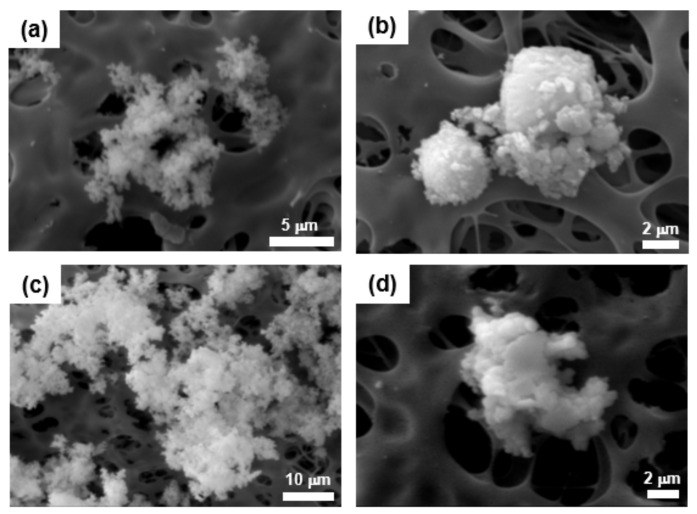
Scanning electron microscopy images of the airborne particles as byproducts the first scrubber maintenance of diffusion process for poly Si and SiO_2_ deposition; (**a**) sample 4, (**b**) sample 5, (**c**) sample 6, (**d**) sample 7.

Figure 1 shows the TEM images of the powder byproducts collected inside the reaction chambers during the first scrubber maintenance of the CVD process for Si_3_N_4_ film deposition. Most of the powder particles were observed to be in both the aggregated and agglomerated formats, with a sub-micrometer size and nearly a spherical and irregular shape. In addition, there were particles that were approximately 10–30 nm in diameter as primary particles, and they were nearly spherical (Figure 1a–c). Figure 2 indicates the SEM images of airborne byproduct particles sampled at the same scrubbers as mentioned above. The airborne particles collected on the filter media surface were also aggregated and/or agglomerated, nearly spherical and irregular, and the size ranged 0.5–35 µm in diameter. In addition, Figure 3 shows the TEM images of the powder byproducts collected inside the reaction chambers during the first scrubber maintenance of the diffusion process for poly Si and SiO_2_ film deposition. The size ranged approximately 50–90 nm, and they were spherical and nearly spherical, based on the primary particle. The particles are likely to be formed by the agglomeration and/or aggregation. For the airborne byproduct particles, it was demonstrated that fine particles, having a size of less than 100 nm, existed on the surface of the aggregates, and the morphology of the particles was mostly spherical or nearly spherical (Figure 4).

### 3.3. Crystal Structure

**Figure 5:**
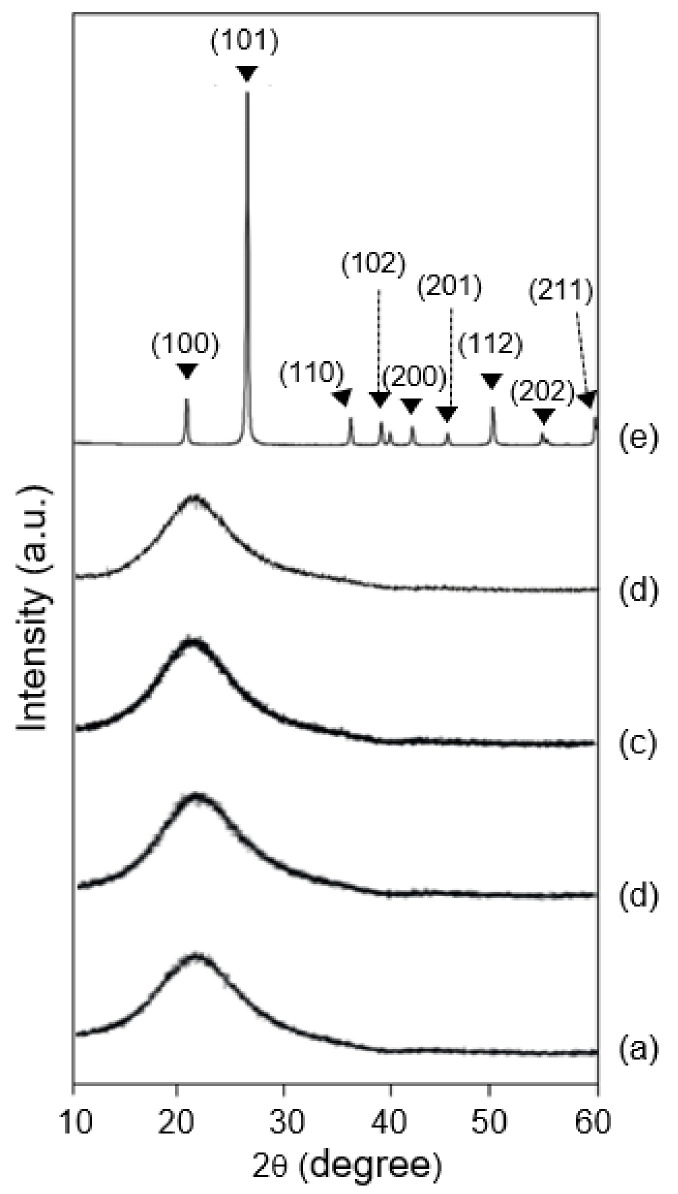
X-ray diffraction patterns of the powder particles as byproducts of the first scrubber maintenance of chemical vapor deposition for Si_3_N_4_ deposition; (a) sample 1, (b) sample 2, (c) sample 3, (d) the pure amorphous silica, and (e) the crystalline silica as comparison samples.

**Figure 6:**
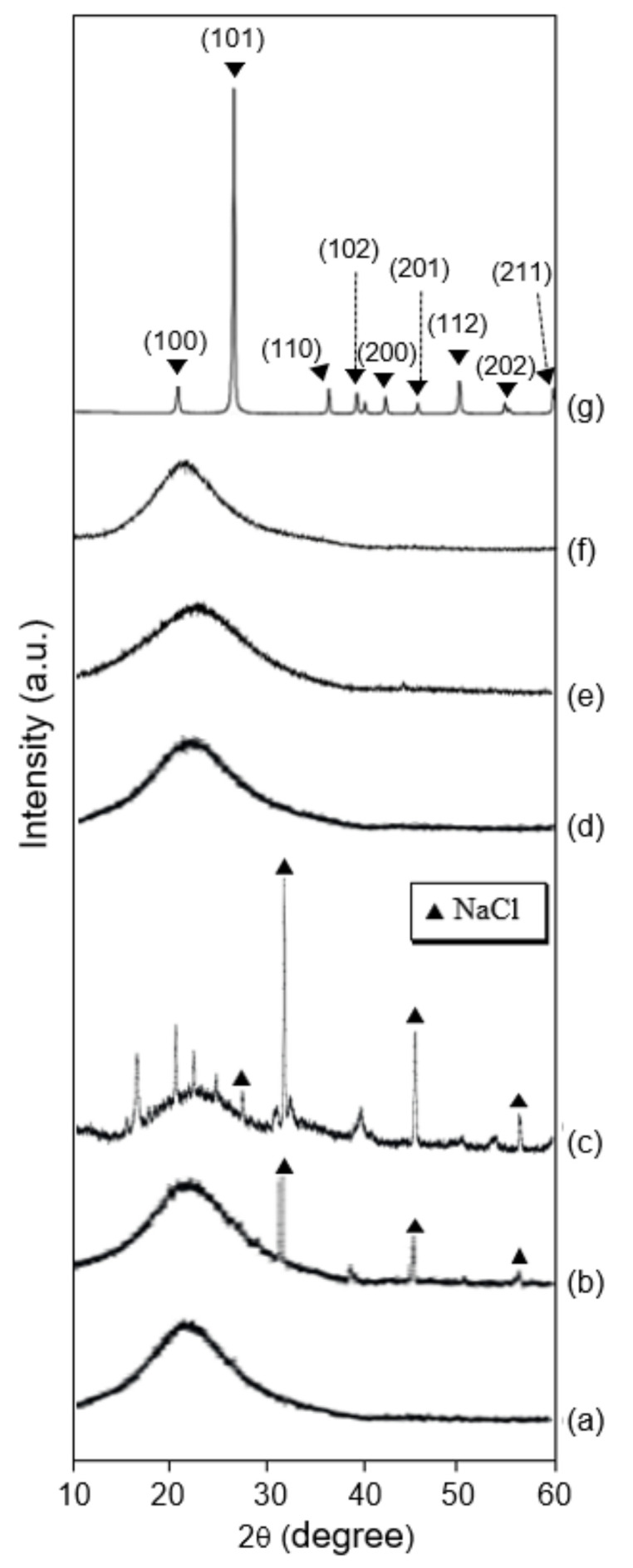
X-ray diffraction patterns of the powder particles as byproducts of the first scrubber maintenance of diffusion process for Poly Si and SiO_2_ deposition; (a) sample 4 (poly Si), (b) sample 5 (poly Si), (c) sample 6 (SiO_2_), (d) sample 7 (SiO_2_), (e) sample 8 (SiO_2_), (f) the pure amorphous silica, and (g) the crystalline silica as comparison samples.

To identify the crystal structure of the powder byproduct particles, XRD analysis was performed. Figure 5 and Figure 6 demonstrate the XRD patterns of the white powder samples collected in the scrubber chamber in the CVD and diffusion processes. For the CVD process, three powder byproducts (Figure 5a–c) exhibited diffraction patterns with a broad and relatively low intensity, at 2θ degrees 21.6–26.7°, which is almost identical to that of pure amorphous silica as a comparative sample (Figure 5d). For the diffusion process, in addition, four powder samples of poly Si (Figure 6a) and SiO_2_ (Figure 6d,e) film deposition steps show almost the same patterns comparted to that of the Si_3_N_4_ step in the CVD process. On the other hand, the other samples of the same steps showed additional sharp patterns, as well as an amorphous silica patterns, and the sharp patterns were similar to that of sodium chloride (NaCl) (Figure 6b,c). This result indicates the Si-containing powder samples are amorphous silica, and they do not contain crystalline silica. On the other hand, the pure crystalline silica of quartz (SiO_2_), as a comparative sample, showed a sharp diffraction peak and relatively high intensity at 2θ degrees 21.7° (Figure 6g). Generally, the quartz has diffraction patterns at 2θ degrees (10° to 60°)–20.85° (100), 26.64° (101), 36.54° (110), 39.46° (102), 42.45° (200), 45.79° (201), 50.13° (112), 54.87° (202), and 59.95° (211) [11,12]. From the above results, the powder byproduct particles collected from the first scrubber of the CVD and diffusion processes were found to be amorphous silica, and so it can be seen that the powder byproducts collected from the production facilities identified in the previous research match the amorphous silica. It was revealed that the powder byproduct particles produced and confirmed in the production process and the exhaust waste gas treatment process were all amorphous silica.

### 3.4. Airborne PM Concentration

**Figure 7:**
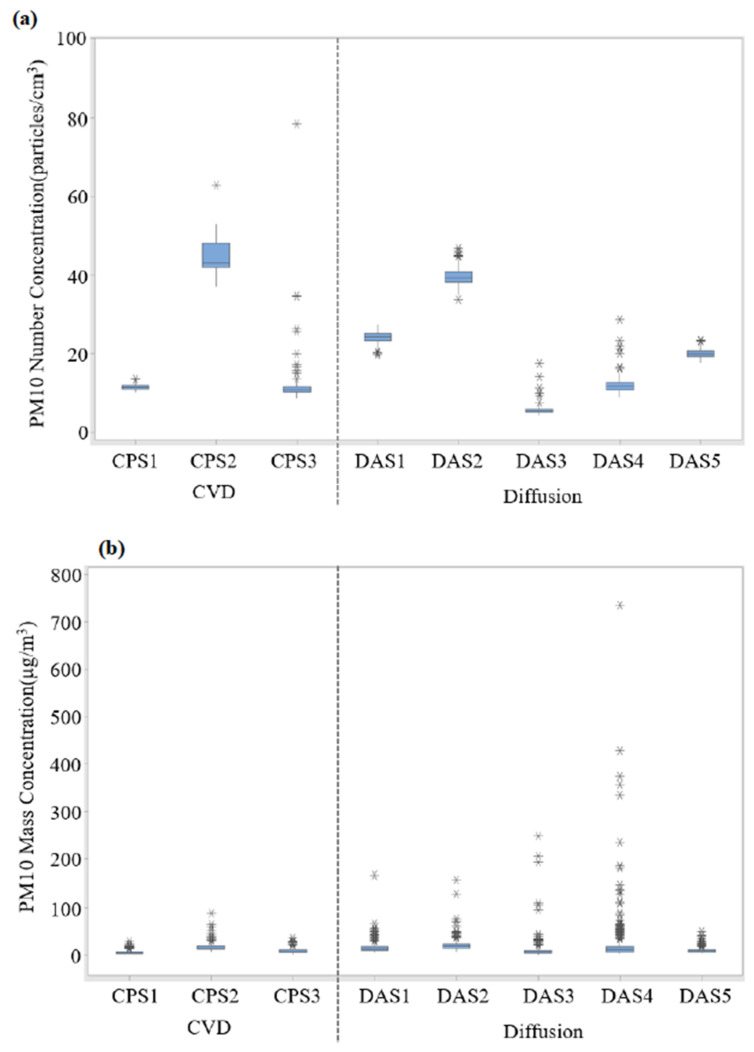
Box plots of (**a**) number and (**b**) mass concentrations of PM10 during maintenance of the first scrubber of chemical vapor deposition and diffusion process.

Figure 7 represents the number and mass concentrations of the airborne PM measured using the OPS around the first scrubber during maintenance. For the CVD process, the number concentrations of PM10 ranged from 8.624–78.466 particles/cm^3^ (avg. range: 11.355–44.537 particles/cm^3^) (Figure 7a). The portions of 0.3–1.0 µm, 1.0–2.5 µm, and 2.5–10 µm particles ranged from 98.68–99.46%, 0.41–1.03%, and 0.10–0.30%, respectively (Supplementary Material Table S1). In addition, the mass concentrations ranged from 1.842–88.084 µg/m^3^ (avg. range: 5.606–16.659 µg/m^3^) (Figure 7b). The portions of 0.3–1.0 µm, 1.0–2.5 µm, and 2.5–10 µm particles ranged from 24.84–49.34%, 3.67–4.83%, and 46.99–70.55%, respectively (Supplementary Material Table S2). On the other hand, for the diffusion process, the number concentrations of PM10 ranged from 4.250–46.680 particles/cm^3^ (avg. range: 5.527–39.627 particles/cm^3^) (Figure 7a). The portions of 0.3–1.0 µm, 1.0–2.5 µm, and 2.5–10 µm particles ranged from 97.10–99.27%, 0.57–1.98%, and 0.01–0.92%, respectively (Supplementary Material Table S1). Moreover, the mass concentrations ranged from 0.939–735.531 µg/m^3^ (avg. range: 9.061–20.979 µg/m^3^) (Figure 7b). The portions of 0.3–1.0 µm, 1.0–2.5 µm, and 2.5–10 µm particles ranged from 9.76–40.66%, 2.79–4.85%, and 55.01–86.68%, respectively (Supplementary Material Table S2). Overall, 0.3–1.0 µm and 2.5–10 µm particles occupied the highest portion of the number and mass concentrations, respectively.

## 4. Discussion

### 4.1. Formative Mechanism

For the diffusion process in the clean room (FAB), it was demonstrated that silica powders, as byproducts, were mainly generated from the SiO_2_ film deposition step around the reaction chamber of the production equipment. Meanwhile, in the first scrubber of the diffusion process in the FS-FAB, it was confirmed that there were silica powders not only in the silicon oxide (SiO_2_) film deposition step, but also in the silicon nitride (Si_3_N_4_) film. The path generation of silica byproduct particles can be estimated in two ways. The first, it is generated by a chemical reaction between the materials including the precursor, such as SiH_4_, Si_2_H_6_, SiCl_2_H_2_, etc., and N_2_O during the CVD and diffusion processes [22,23,24]. The second, it is also generated by hydrolysis and the thermal oxidation of the remaining chemicals used in the reaction in the scrubber. Since SiH_2_Cl_2_ and N_2_O are only used as process gases, the materials that can be generated as byproducts are SiO_2_, HCl and N_2_. In addition, SiO_2_, HCl and H_2_ can be generated when SiH_2_Cl_2_ is exposed to moisture (H_2_O).

### 4.2. Elemental Component and Size

In the first scrubber of the CVD process, including Si_3_N_4_ film deposition, it can be assumed that the Si and F elements are due to process gases (such as SiH_4_, SiCl_2_H_2_) and the chamber cleaning gas of NF_3_, respectively. Meanwhile, for the diffusion process, in addition to Si as the main element, F, Ni, Na, Mg, Al, K, and Ca were also intermittently detected as minor. The F element is estimated to be caused by the decomposition of NF_3_ as the chamber cleaning gas. Meanwhile, it is assumed that the other elements are due to leaching from the chamber surface, and/or cross-contamination by air circulation from other vicinal processes or outdoor air. Generally, the concentration levels and elemental components of PM in a semiconductor FS-FAB are partially affected by those of the outdoor air [21]. In addition, it is known that the principal element of PM at the urban roadside is Ca. Meanwhile, Al and K are commonly detected in various sites, such as the urban roadside, urban background, and rural area [25]. In fact, these elements are the most frequently observed in various ambient air studies [26,27,28]. The powder and airborne particle size, based as a primary particle for the semiconductor processes such as CVD and diffusion during maintenance of the first scrubbers, can be summarized as follows: powder, diffusion (50–90 nm) > CVD (10–30 nm); airborne particle, CVD (0.8–35 µm) > diffusion (0.2–10 µm). The data suggest that nanoparticles are formed and exist in the inner side of the chamber, and agglomerated and/or aggregated with time.

### 4.3. Airborne Concentration and Peak Exposure

While the clean room (or FAB) is strictly controlled by particles in the air, the FS-FAB is managed relatively loosely because it has no product defect effect, thus it is partially affected by fine particles in outdoor air because it is not managed by the ultra-low penetration air filter (removal efficiency of airborne particles based on 0.1 µm diameter: 99.99995%). However, the removal efficiencies of airborne particle of the pre- and medium filters in the out air handling unit system for the FS-FAB are more than 80 and 90%, based on a 10 and 0.5 µm diameter, respectively. Thus, in general, the airborne particle concentration in FS-FAB is lower than that of the general office and outdoor air [21]. In fact, in this study, the average ranges of the number and mass concentration of PM10 during maintenance of all the first scrubbers are 5.527–44.537 particles/cm^3^ and 5.606–20.979 µg/m^3^, respectively. These data are much lower than outdoor air and similar to the office [21]. The current threshold limit values (TLVs) for amorphous silica (SiO_2_) are 6 mg/m^3^ [time weighted average (TWA) concentration for up to 10 hr workday during a 40 hr workweek], as recommended by the National Institute for Occupational Safety and Health (NIOSH) [29]. In addition, respirator recommendations of NIOSH are as follow: (Assigned Protection Factor (APF) = 5) any quarter mask respirator up to 30 mg/m^3^; (APF = 10) any particulate respirator equipped with an N95, R95, or P95 filter (including N95, R95, and P95 filtering facepieces) except quarter-mask respirators up to 60 mg/m^3^. Herein, APF means the workplace level of respiratory protection that a respirator or class of respirators is expected to provide to employees when the employer implements a continuing, effective respiratory protection program, as specified by this section. Since the measurement and analysis methods are not the same, there is a limit to clearly comparing the exposure concentration to amorphous silica. Nevertheless, the airborne concentration of SiO_2_ particles during the first scrubber maintenance was extremely low compared to those of the NIOSH exposure standard. Due to local exhaust ventilation systems, the byproduct particles generated from the first scrubber are rarely exposed to ambient air during maintenance. However, peak exposure of SiO_2_ byproduct particles has been confirmed several times during maintenance tasks of the first scrubbers, i.e., step 3: remove the powder particles in the scrubber chamber and duct by using a vacuum cleaner; and step 4: remove the powder particles on the inside and outside surface of the scrubber chamber by using the vacuum cleaner and a clean wiper (Supplementary Material Figure S3). Therefore, the maintenance workers should be careful not to scatter the powder byproducts, and the respiratory protective equipment (RPE) should be worn through sufficient training, including a fit-test when workers are put on the RPE before the maintenance. Overall, the potential inhalation exposure to amorphous SiO_2_ particles as the byproduct during maintenance among workers would be very low because the workers wear respirators and local exhaust ventilation systems, with an exterior-type hood for controlling airborne emission of the SiO_2_ byproduct particles, are operating. There is still a lack of data available from studies so far on human health impacts. Therefore, continued research in conditions similar to the exposure of amorphous SiO_2_ particles in the working environment will make it possible to confirm the toxicity more clearly.

### 4.4. Health Effects

Many studies have been conducted on the health effects of SiO_2_ particles, and the health effects of silica are dependent on the crystalline structure [13,14,15]. Crystalline silica particles may cause pulmonary fibrosis responses, and are a known carcinogen (Group 1) recognized by the International Agency of Research on Cancer (IARC), whereas, amorphous silica is considered to be less toxic. However, the crystal structure of the SiO_2_ byproducts generated from the first scrubbers in the CVD and diffusion processes was amorphous, and amorphous silica has not been implicated in causing pulmonary fibrosis or cancer. Amorphous silica, such as fumed silica, may cause irreversible lung damage in some cases, but is not associated with the development of silicosis. Children, asthmatics of any age, those with allergies, and the elderly (all of whom have a reduced lung capacity) can be affected in less time [30]. To evaluate the health risks, the exposure and hazard of the agent to which the worker is exposed should be considered. The amorphous SiO_2_ particles as byproducts have not yet been shown to be carcinogenic, mutagenic, or exert reproductive toxicity. Size is a critical parameter for the distribution of particles in the human body. Generally, the toxicity of different sized particles can differ in different regions of the lungs. Nanoparticles are very capable of reaching the fragile structure of the alveoli; their deposition in the alveoli increases with a decreasing diameter until 20 nm [19]. Thus, there is the potential for particles to deposit themselves in the alveoli throughout the airway. In addition, the average ranges of the mass concentration of airborne PM10 during maintenance of all first scrubbers are 5.606–20.979 µg/m^3^, an extremely low level. Although the amorphous silica particles, as byproducts, generated from first scrubber show a low toxicity and low exposure concentration, it is necessary to make continuous improvements to the process and work environment because the influence of chronic low-level exposure cannot be excluded. The potential inhalation exposure to amorphous silica as byproducts during the maintenance of first scrubber among workers would be very low because the workers wear half-mask respirators with a particulate filter and multi gases-vapor cartridge, and local ventilation systems are operating.

## 5. Conclusions

This study focused on the hazard identification and exposure measurement of powder and airborne particles as byproducts collected from the first scrubber of the chemical vapor deposition and diffusion processes, which used silicon containing compounds as precursor materials during maintenance in a semiconductor sub fab. For all powder and airborne particle samples, O and Si were mainly detected, which indicates the powder byproduct is silicon oxide. The byproduct particles were spherical and/or nearly spherical, and the particle size range 10 to 90 nm based on primary particles, and most of the particles were usually agglomerated within a particle size range, from approximately 100 nm to 35 µm. In addition, most of the powder samples exhibited diffraction patterns with a broad and relatively low intensity at 2θ degrees 21.6–26.7°, which is similar to that of pure amorphous silica. The above results show the byproduct particles are amorphous silica, which are considered a less toxic foam compared to crystalline silica. During the maintenance of the first scrubbers, the number and mass concentrations of PM10 ranged from 4.250–78.466 particles/cm^3^ and 0.939–735.531 µg/m^3^, respectively. In addition, 0.3–1.0 and 2.5–10 µm particles occupied the highest portion of the number and mass concentrations, respectively. On the other hand, several peak exposure patterns were observed at specific steps during maintenance task, which are the processing removal of powder byproducts on the inner chamber, and cleaning the chamber using a vacuum cleaner and a clean wiper. Overall, the exposure concentration of the amorphous SiO_2_ byproduct particles during maintenance is extremely low, and potential human effects of the particles are very low compared to that of crystalline silica. Nevertheless, it should be important to operate a system that can thoroughly comply with the process of worker training (including a fit-test) and standard operating procedures, as well as continuous infrastructure improvements in the semiconductor working environment, because the influence of chronic low-level exposure cannot be excluded.

## Figures and Tables

**Table 1:** Elemental compositions of powder and airborne particles as byproducts generated during maintenance of the first scrubbers of chemical vapor deposition and diffusion processes, and the chemicals used in the processes.

Process	Film	Chemicals Used	Scrubber (Sample No)	Elemental Composition ^a^	
				Powder ^b^	Airborne Particle ^c^
Chemical Vapor Deposition	Si_3_N_4_	SiH_4_, SiCl_2_H_2_, NH_3_	CPS1 (1)	O, Si, (F)	ND
			CPS2 (2)	O, Si	O, Si, (F)
			CPS3 (3)	O, Si, (F)	O, Si
Diffusion	Poly Si	SiH_4_, Si_2_H_6_, Si_3_H_8_	DAS1 (4)	O, Si, (F)	O, Si
			DAS2 (5)	O, Si, (F)	O, Si (Na, Mg, Al, K, Ca)
	SiO_2_	SiCl_2_H_2_, N_2_O	DAS3 (6)	O, Si, (Ni, Na)	O, Si
			DAS4 (7)	O, Si	O, Si
			DAS5 (8)	O, Si, (Ni)	ND

Notes: ^a^ Carbon (C), chlorine (Cl) and/or copper (Cu) among elemental components were omitted because carbon tape, PVC components and/or copper grid as a background was used for SEM-EDS or TEM-EDS analysis, respectively; ^b^ TEM-EDS: transmission electron microscopy equipped with energy dispersive X-ray spectroscopy; ^c^ SEM-EDS: scanning electron microscopy equipped with energy dispersive X-ray spectroscopy; elements in parentheses are minor components with low intensity from transmission electron microscopy and scanning electron microscopy equipped with energy dispersive X-ray spectroscopy.

## Data Availability

Not applicable.

## Supplementary Materials

The following supporting information can be downloaded at: https://www.mdpi.com/article/10.3390/ijerph19031791/s1, Table S1: Number concentration distributions according to the particle size, e.g., 0.3–1.0, 1.0–2.5, and 2.5–10 µm, during the maintenance of the first scrubber of the chemical vapor deposition and diffusion processes, Table S2: Mass concentration distributions according to the particle size, e.g., 0.3–1.0, 1.0–2.5, and 2.5–10 µm, during the maintenance of the first scrubber of the chemical vapor deposition and diffusion processes, Figure S1: Mass concentration of airborne byproduct particles according to the maintenance task of the first scrubber (DSA4) of the diffusion process.
