# Supplementary material for: Physicochemical Characteristics and Occupational Exposure of Silica Particles as Byproducts in a Semiconductor Sub Fab
Source: Int J Environ Res Public Health. 2022 Feb 4;19(3):1791. doi: 10.3390/ijerph19031791 (PMC8835547; doi:10.3390/ijerph19031791)
Supplement: Supplementary file 1 [file ijerph-19-01791-s001.zip › ijerph-1512707-supplementary.pdf]

Supplementary Materials

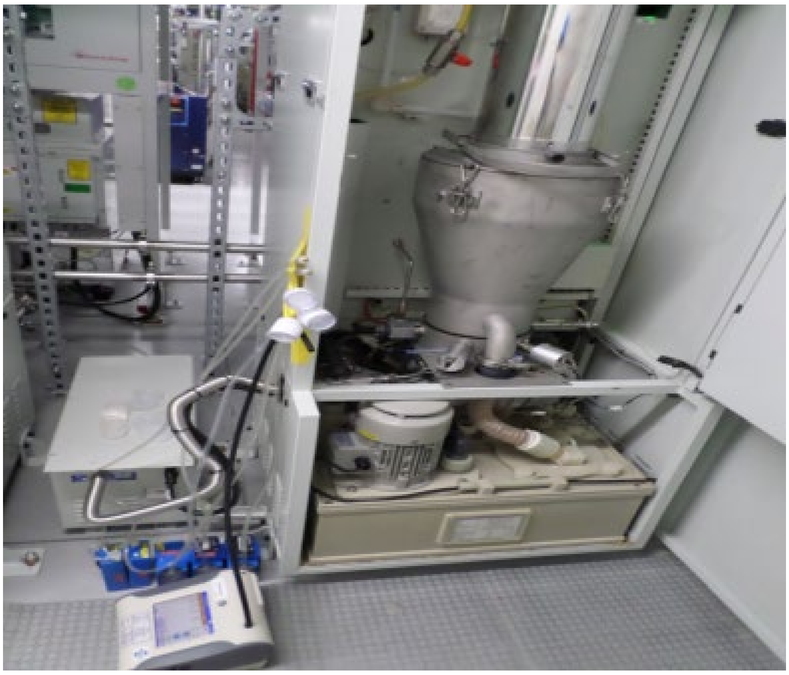

Figure S1. Image of number and mass concentration measurement of airborne particles using optical particle sizer during maintenance of scrubbers.

Sample No	Powder (TEM image)	Airborne particle (SEM image)
CPS1 (1)	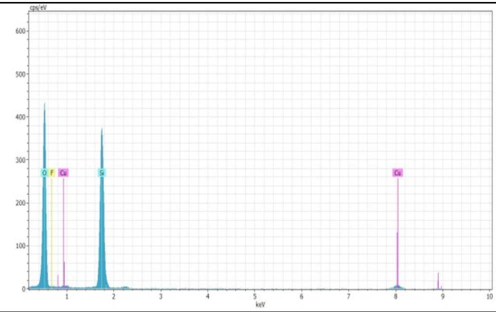	ND
CPS2 (2)	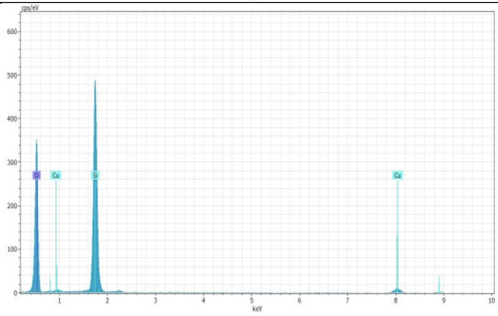	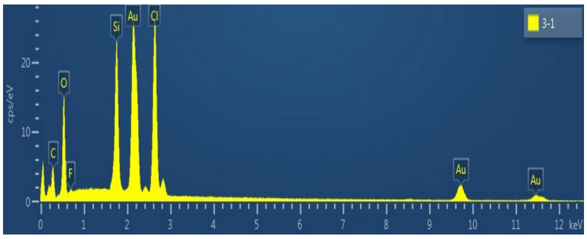
CPS3 (3)	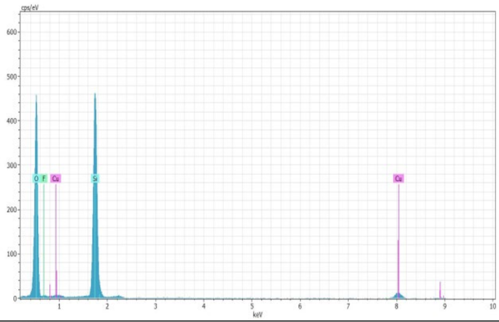	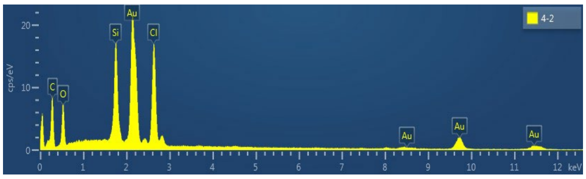
DAS1 (4)	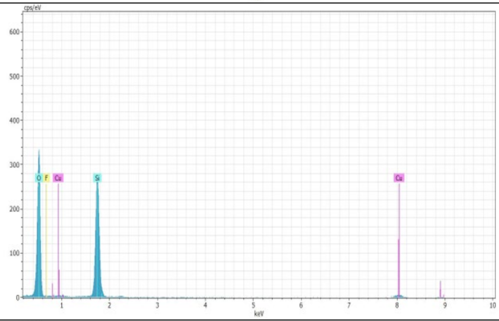	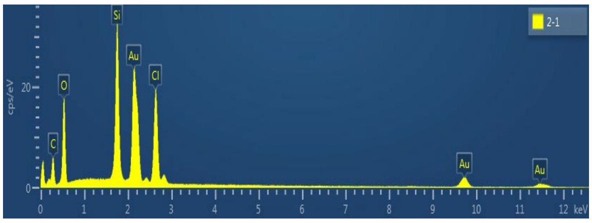
DAS2 (5)	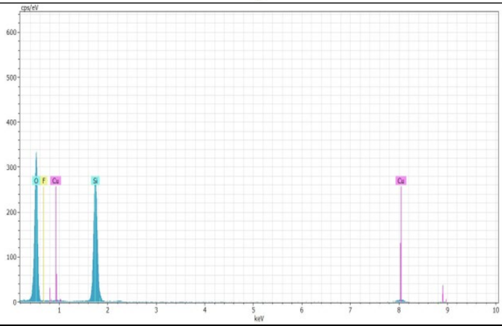	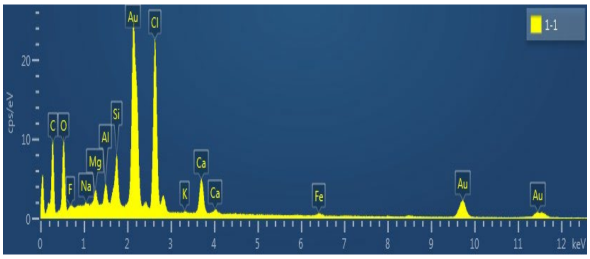

DAS3<br>(6)	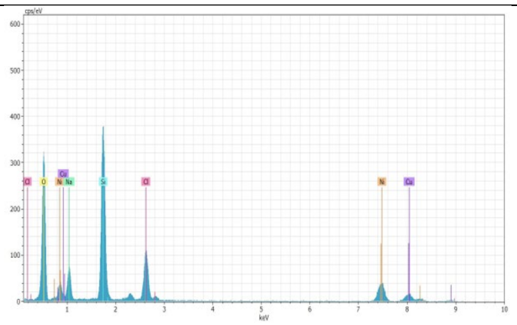	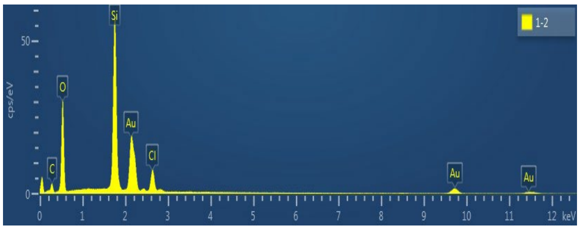
DAS4<br>(7)	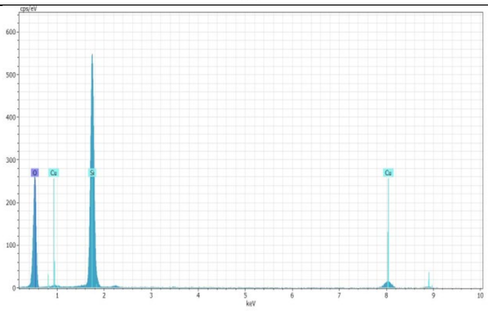	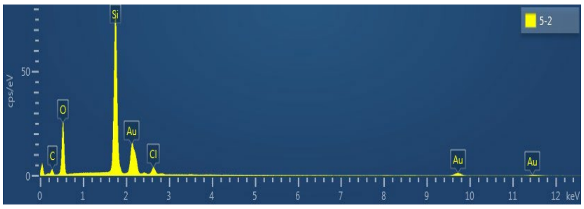
DAS5<br>(8)	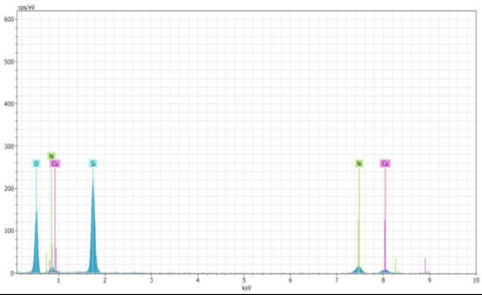	ND

Figure S2. Energy dispersive spectroscopy spectra of elemental compositions of powder and airborne particles in Table 1.

Table S1. Number concentration distributions according to the particle size, e.g., 0.3-1.0, 1.0-2.5, and 2.5-10 μm during the maintenance of first scrubber of chemical vapor deposition and diffusion processes.

Process	Scrubber	Number concentration distribution (%)		
		0.3-1.0 μm	1.0-2.5 μm	2.5-10 μm
Chemical Vapor Deposition	CPS1	99.129	0.710	0.161
	CPS2	99.485	0.413	0.101
	CPS3	99.676	1.025	0.299
Diffusion	DAS1	98.898	0.878	0.224
	DAS2	99.247	0.565	0.188
	DAS3	97.102	1.977	0.920
	DAS4	97.858	1.349	0793
	DAS5	99.266	0.590	0.014

Table S2. Mass concentration distributions according to the particle size, e.g., 0.3-1.0, 1.0-2.5, and 2.5-10 μm during the maintenance of first scrubber of chemical vapor deposition and diffusion processes.

Process	Scrubber	Number concentration distribution (%)		
		0.3-1.0 μm	1.0-2.5 μm	2.5-10 μm
Chemical Vapor Deposition	CPS1	37.711	4.828	57.461
	CPS2	49.335	3.672	46.994
	CPS3	24.836	4.618	70.546
Diffusion	DAS1	30.493	4.848	64.658
	DAS2	34.772	3.546	61.682
	DAS3	9.758	3.558	86.684
	DAS4	11.312	2.793	85.895
	DAS5	40.661	4.331	55.009

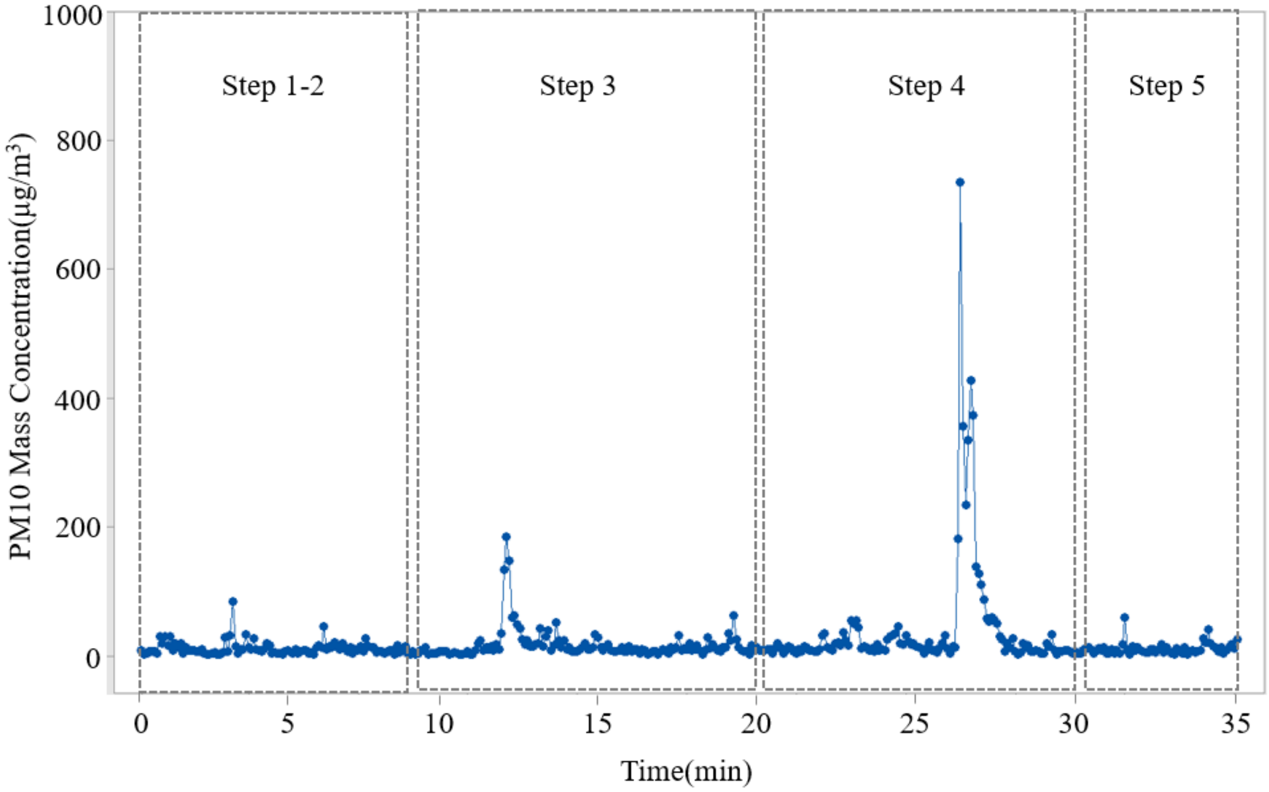

Figure S3. Mass concentration of airborne byproduct particles according to maintenance task of first scrubber (DSA4) of diffusion process.
